# Plant regeneration and transformation of *Trachyspermum ammi* using *Agrobacterium tumefaciens* and zygotic embryos

**DOI:** 10.1186/s43141-021-00173-8

**Published:** 2021-05-11

**Authors:** Masoumeh Nomani, Masoud Tohidfar

**Affiliations:** 1 Department of Agronomy and Plant breeding - College of Aburaihan, University of Tehran, Tehran, Iran; 2 Department of Plant Biotechnology – College of Life Science and Biotechnology, Shahid Beheshti University, Daneshjou Boulevard, Tehran, 19839-63113 Iran

**Keywords:** Plant growth regulators, HPLC, Zygotic embryo, Transgenic, PCR, Southern blot

## Abstract

**Background:**

*Trachyspermum ammi* is one of the key medicinal plant species with many beneficial properties. Thymol is the most important substance in the essential oil of this plant. Thymol is a natural monoterpene phenol with high anti-microbial, anti-bacterial, and anti-oxidant properties. Thymol in the latest research has a significant impact on slowing the progression of cancer cells in human. In this research, embryos were employed as convenient explants for the fast and effectual regeneration and transformation of *T. ammi*. To regenerate this plant, Murashige and Skoog (MS) and Gamborg's B5 (B5) media were supplemented with diverse concentrations of plant growth regulators, such as 6-benzyladenine (BA), 1-naphthaleneacetic acid (NAA), 2,4-dichlorophenoxyacetic acid (2,4-D), and kinetin (kin). Transgenic *Trachyspermum ammi* plants were also obtained using *Agrobacterium*-mediated transformation and zygotic embryos explants. Moreover, two *Agrobacterium tumefaciens* strains (EHA101 and LBA4404) harboring pBI121-TPS2 were utilized for genetic transformation to *Trachyspermum ammi*.

**Results:**

According to the obtained results, the highest plant-regeneration frequency was obtained with B5 medium supplemented with 0.5 mg/l BA and 1 mg/l NAA. The integrated gene was also approved using the PCR reaction and the Southern blot method. Results also showed that the EHA101 strain outperformed another strain in inoculation time (30 s) and co-cultivation period (1 day) (transformation efficiency 19.29%). Furthermore, HPLC method demonstrated that the transformed plants contained a higher thymol level than non-transformed plants.

**Conclusions:**

In this research, a fast protocol was introduced for the regeneration and transformation of *Trachyspermum ammi*, using zygotic embryo explants in 25–35 days. Our findings confirmed the increase in the thymol in the aerial part of *Trachyspermum ammi.* We further presented an efficacious technique for enhancing thymol content in *Trachyspermum ammi* using *Agrobacterium*-mediated plant transformation system that can be beneficial in genetic transformation and other plant biotechnology techniques.

## Background

Medicinal plants are the most preferred topic for the plant biotechnology researcher, because of their pharmaceutical combination. In many parts of the world, utilization of medicinal plants is of highly important because of health care [1]. *Trachyspermum ammi* is a traditional and medicinal herb, which is highly effective in curing various human and animal diseases. This plant is also of nutritional and medicinal importance.

Achievements in in vitro regeneration and reproduction of medicinal plants rely on many elements [2]. For example, the shortest and most efficient protocol for explant propagation is of great value in tissue culture and gene transfer methods. Several studies have been conducted on the regeneration of *T. ammi* with different explants, all of which took a long time to regenerate [3–7]. However, as far as we know, there is no survey about tissue culture by means of zygotic embryo explants for this plant. The success of the embryo propagation technique depends on isolating the embryo and determining the appropriate culture media and growth regulators. Auxin and cytokinin growth regulators have a significant role in growth quality in embryos. In the former research works, the efficacy of auxins and cytokinins in regeneration of several medicinal plants has been studied [8–10]. Biotechnology by techniques, such as cell and tissue culture, genetic engineering, molecular markers, metabolic engineering, and gene overexpression have proven to be able to enhance the effectiveness of medicinal plants as a renewable resource for drug production [11, 12].

Terpenoids are one of the most diverse classes of natural products. Based on *Agrobacterium*-mediated genetic evolution, several plant metabolic engineering strategies are promising to regulate the biosynthesis of medicinal terpenoids such as overexpression of terpenoid biosynthesis pathway genes in plants and suppression expression of competitive metabolic pathways [13]. During the recent years, using overexpression, several monoterpene and sesquiterpene synthases have been changed to generate new monoterpenes, and sesquiterpenes that were present in floral and green tissues [14, 15]. Moreover, much attention has been lately paid to the synthesis pathways of terpenes. Most terpenes in herbs are involved in producing secondary metabolites [16]. TPS2 is one of the most important synthesizing enzymes of monoterpenes [17]. Previous studies on *T. vulgaris* have demonstrated that γ-terpinene is a major precursor in the aromatic monoterpene pathway leading to production of p-cymene, thymol, and carvacrol by the terpene synthase 2 [17, 18]. However, nowadays, production of the most secondary metabolites is possible through manipulation of synthesis pathways in medicinal plants [19, 20]. Thymol is a valuable and the main essential oil existing in *T. ammi* [21]. Many of the therapeutic properties of this plant are relevant to thymol [22]. In brief, transgenic plants’ procedure consists of transferring the selected gene into an extremely totipotent explant using *Agrobacterium* strains and development of regenerated plants [23]. On the other hand, optimization of gene transfer process plays an important role in the success of plant transformation. The transformation of the *GUS* gene along with *Agrobacterium* has been carried out in some medicinal plants [24–26].

Previous studies reported that overexpression of *TYDC2* in the opium poppy led to an increase in morphine, codeine, and the baine alkaloids in the transgenic plants compared to the non-transgenic plants [27]. The findings of previous research on overexpression of codeinone reductase in *Papaver bracteatum* revealed the production of Codeine (0.04% dry wt) and morphine (0.28% dry wt) in the transgenic hairy root [28]. It was demonstrated that overexpression of *SmMYC2* in *Salvia miltiorrhiza* resulted in the production of phenolic acids [29]. Jiang carried out overexpression of *AaWRKY1* in *Artemisia annua* and showed that *AaWRKY1* increased the content of artemisinin in this plant [30]. In another major study on *Artemisia annua*, it was found that overexpression of the cytochrome P450 monooxygenase and cytochrome P450 reductase genes can increase artemisinin level [31]. In this report, we described the application of an *Agrobacterium*-mediated transformation system to a *T. ammi* (Shaheideh Yazd). This method combines the use of the zygotic embryo as an explant and the ability of *Agrobacterium* for transformation cells. Zygotic embryo explant is superior to other regeneration systems due to its independent genotype. The zygotic embryo has been accepted as an adequate explant for tissue culture and genetic transformation in many medicinal plants [32–35]. Since, there have been difficulties with the regeneration of plants from *Agrobacterium*-infected zygotic embryo, the *Trachyspermum ammi* transformation system is established. Therefore, this research aimed to provide a fast procedure to increase thymol using zygotic embryos explant for overexpressing *TPS* gene in *T. ammi*. This presented procedure can be beneficial for the genetic transformation of other genes and further biotechnology studies.

## Methods

### Seed disinfection, preparation of explants, callus induction, and regeneration

The mature seeds of *T. ammi* (ecotype of Shaheideh Yazd) were provided from the Research Institute of Forests and Rangelands of Iran. For seed disinfection, they were first submerged in 70% ethyl alcohol and Tween-20 for 60 s. Next, they were disinfected with 1.5% sodium hypochlorite (SH) (w/v) for 3 min by gentle agitation. Then, sterilized seeds were rinsed at least three times with sterile double-distilled water (each for time lasting 5 min). Afterwards, the seeds were dried on a sterile paper and were located in 50 ml conical tubes containing autoclaved water for 24 h in a growth chamber at 25±2°C under 16/8 h (light/darkness) photoperiod. After 24 h, the seeds were placed on a filter paper for 3 min in a sterile situation. Afterwards, embryos were separated from swollen seeds and used as explants. Isolated embryos were cultured on MS [36] and B5 [37] media supplemented with different concentrations of NAA, BA, 2,4-D, KIN, 3% (w/v) sucrose, and 0.7% (w/v) agar. Before autoclaving (121°C, 1.04 kg cm^2^, 20 min) of media, pH was adjusted to 5.7–5.8. The Petri dishes were incubated in the growth chamber at 25±2°C with the light intensity of about 2000 Lux provided by white fluorescent lamps and photoperiod of 16/8 h. Furthermore, to assess the effect of growth regulators on zygotic embryo propagation, different of various concentrations of NAA (0.5, 1, 2), BA (0, 0.5, 1), 2,4-D (0.5, 1, 2), and KIN (0.2, 0.5, 1) on B5 (salts + vitamins) or MS (salts + vitamins) media were applied (Table 1).

**Table 1:** Effect of Murashige and Skoog (MS) and Gamborg’s B5 (B5) media with diverse concentrations of plant growth regulators, 6-benzyladenine (BA), 1-naphthaleneacetic acid (NAA), 2,4-dichlorophenoxyacetic acid (2,4-D), and kinetin (KIN) to regenerate *Trachyspermum ammi* by zygotic embryos

	Treatment	(mg/l)		Medium	Callusinduction	Fresh Weightof callus	Dry Weightof callus	Height of shoot	Regeneration	Average numberof shoots
BA	NAA	2,4-D	KIN		(%)	(mg)	(mg)	(cm)	(%)	
0	0.5	0	0	B5	68^c^	0.47	0/03	7.65	62.7^c^	4.7^b^
0	1	0	0	B5	56^d^	0.41	0.034	7.21	65.5^c^	4.01^bc^
0	2	0	0	B5	52^d^	0.44	0.032	6.97	77.4^bc^	4.12^bc^
0.5	0.5	0	0	B5	84^b^	0.48	0.031	9.54	83.4^b^	4.23^bc^
0.5	1	0	0	B5	98^a^	0.53	0.034	10.23	92.6 ^a^	5.9^a^
0.5	2	0	0	B5	80^bc^	0.41	0.029	9.12	89.97^b^	4.8^b^
1	0.5	0	0	B5	88^b^	0.43	0.031	9.93	76.6^bc^	4.9^b^
1	1	0	0	B5	74^bc^	0.37	0.027	9.17	80.75^bc^	4.54^bc^
1	2	0	0	B5	44^e^	0.29	0.018	9.19	68.5^c^	3.7^c^
0	0	0.5	0.2	MS	72^bc^	0.31	0.028	7.76	58.8^d^	3.87^c^
0	0	0.5	0.5	MS	64^c^	0.37	0.021	7.98	56.2^d^	3.54^c^
0	0	0.5	1	MS	56^d^	0.39	0.023	6.74	49d^e^	2.5^cd^
0	0	1	0.2	MS	96^a^	0.41	0.029	9.97	82.5^b^	3.9^c^
0	0	1	0.5	MS	88^b^	0.47	0.032	7.54	79.9^bc^	3.76^c^
0	0	1	1	MS	78^bc^	0.31	0.029	6.98	68.2^c^	1.97^d^
0	0	2	0.2	MS	52^d^	0.38	0.021	7.12	61.7^d^	3.81^c^
0	0	2	0.5	MS	44^e^	0.33	0.027	6.34	51.3^de^	2.87^cd^
0	0	2	1	MS	31^f^	0.27	0.018	5.53	41.1^e^	1.89^d^

^a–f^Means with different superscripts within the same column differ significantly (*P* ≤ 0.05) using Duncan

After culturing the embryos, calluses were appeared after 8–10 days. The callus induction was determined as following formula:

$$ \mathrm{Callus}\ \mathrm{induction}=\frac{\mathrm{Number}\ \mathrm{of}\ \mathrm{calluses}\ }{\mathrm{Total}\ \mathrm{number}\ \mathrm{of}\ \mathrm{zygotic}\ \mathrm{embryos}}\times 100 $$

Afterwards, callus was transmitted to a fresh medium with the same growth regulators for regeneration, and after 12 days, the percentages of plantlets were counted as following formula:

$$ \mathrm{Regenertion}=\frac{\mathrm{Number}\ \mathrm{of}\ \mathrm{plantlet}}{\mathrm{Total}\ \mathrm{number}\ \mathrm{of}\ \mathrm{calluses}}\times 100 $$

Following the determination of calluses fresh weight, we dried them in an oven at 60°C for 24 h and their dry weights were measured. Additionally, after regeneration, the roots of the plantlets were rinsed with tap water. Subsequently, to maintain humidity, the plantlets were placed in plastic pots containing a mixture of sterilized soil vermiculite, garden soil, and perlite with plastic covers for 10 days. Eventually, the plantlets were cultured in larger pots and placed in the greenhouse.

### Bacterial strain and plasmid

The transgenic *T. ammi* was prepared using hypocotyl explants [38], but this method produces a small number of plants and is time-consuming. So, another procedure was used to produce more transgenic plants in a shortest period of time. We also used *Agrobacterium tumefaciens* EHA101 and LBA4404 with the genetic construct of pBI121-TPS2 as well as zygotic embryos. This vector contains the *TPS* and *neomycin phosphotransferase (nptII)* gene, as a selectable marker. To optimize transformation factors, a strain of *Agrobacterium* harboring the binary vector pBI121 possessing the *GUS* gene was also used.

### Transformation, co-cultivation, and regeneration of transgenic plants

To determine the appropriate level of kanamycin for gene transfer, zygotic embryos were isolated (as explained in the previous section) and placed on the media (best chosen media based on tissue culture) with diverse levels of kanamycin (0, 5, 10, 15, 20, 25, 30, 50, and 75 mg/l). Kanamycin was added following the preparation of autoclaved selective medium and after cooling (40–50°C) in a laminar flow hood. In the transformation step, a single *Agrobacterium* colony grown on LB solid medium with kanamycin (50 mg/l) was cultured for 48 h and then transferred to the conical tube containing LB liquid with suitable antibiotics and incubated overnight in an incubator at 28°C on a rotary shaker (OD600 = 0.4–0.6). Additionally, in transformation processing, two vectors, pBI121-TPS and pBI121, were used. In the laminar hood, the embryos were separated from seeds (described previous) and immersed in bacterial suspension in 15-ml conical tubes several times (30 s, 1 min, 2 min, 3 min, and 5 min) with gentle shaking. Then, they were put on sterile paper to get dried after inoculation. In the next step, the explants were placed on co-cultivation medium (best chosen media with antibiotics) at diverse times (1 and 2 days). Finally, they were cultured on callus induction medium adding 15 mg/l kanamycin and 180 mg/l cefotaxime to inhibit *Agrobacterium* strains’ growth and were placed in a growth chamber.

### Molecular analysis, histochemical *GUS* assay, and RT-PCR

In the present research, total genomic DNA of the plant was extracted from leaves of the transformed and non-transformed herbs to be used for PCR and subsequently verify the presence of genes in transgenic herbs (Table 2). The PCR status was identified as follows: initial denaturation for 5 min at 94°C, then 35 subsequent cycles of denaturation runs for 1 min at 94°C, annealing at 60°C (*GUS*), 61°C (*TPS2*), and 62°C (*nptII*) genes for 1 min then extension at 72°C for 45 s and the last extension at 72°C for 5 min. *GUS* expression was also determined for randomly selected transformed plants. The histochemical *GUS* test was performed based on Jefferson instruction [39]. Small samples were isolated from leaves of the transformed and non-transformed plants and immersed in 1.5-ml tubes. Then, X-gluc (5-bromo-4-chloro-3-indolyl-β-glucuronidase) buffer was added into the tubes and kept at a temperature of 37°C (room temperature) for 24 h. Afterward, the solution was taken out of the tube and the plants immersed in 70% ethanol for 4 h. Finally, the samples were observed under a binocular stereomicroscope.

**Table 2:** Nucleotide sequences of primers used in PCR

Gene	Primer sequences 5′ to 3′
*Gus*	F: 5′ ACCTCGCATTACCCTTACGCTGAA 3′ R: 5′ AATCGCCGCTTTGGACATACC 3′
*Tps2*	F: 5′ ACTCGTCTCCGTCCTATC 3′ R: 5′ CGTCCTTCGTATTCTCAC 3′
*nptII*	F: 5′ GTCATCTCACCTTGCTCCTGC 3′ R: 5′ AAGAAGGCGATAGAAGGCG 3′
*Vir G*	F: 5′ ATGATTGTACATCCTTCACG 3′ R: 5′ TGCTGTTTTTATCAGTTGAG 3′

The total RNA from leaves of transformed plants was extracted using trizol (Invitrogen, USA) for final verification of the transformed plants and stable expression of *GUS* gene. After treatment with DNase, the first cDNA strand was produced by a cDNA synthesis kit (Eurex) and Oligo-dT primer. Finally, two strands of cDNA were synthesized in a thermal cycler (Bio-Rad) by specific primers.

### DNA gel-blot analysis and HPLC

Ten micrograms of DNA were digested with an *Eco*RI enzyme that cuts only one site within T-DNA. Then, digested DNA was loaded onto the 0.8% (w/v) agarose gel and blotted to a positively charged nylon membrane according to the instruction (HAYBOND N+, Amersham, Little Chalfont, UK). The probe corresponding to a PCR product of the *TPS2* target gene was produced and detection was done by DIG detection kit (Boehringer, Mannheim, Germany). Air-dried leaves and inflorescence of transformed and non-transformed herbs were grounded and extracted. Afterward, water liquid chromatography was conducted to determine thymol content [40].

### Statistical analysis

The treatments were conducted as factorial experiments based on a completely randomized design (CRD) with 3 replications. Mean comparisons were performed through Duncan’s multiple range test (*P* < 0.05) using SAS software (version 9.3). Excel was used for plotting.

## Results

### Callus induction and regeneration

**Fig. 1:**
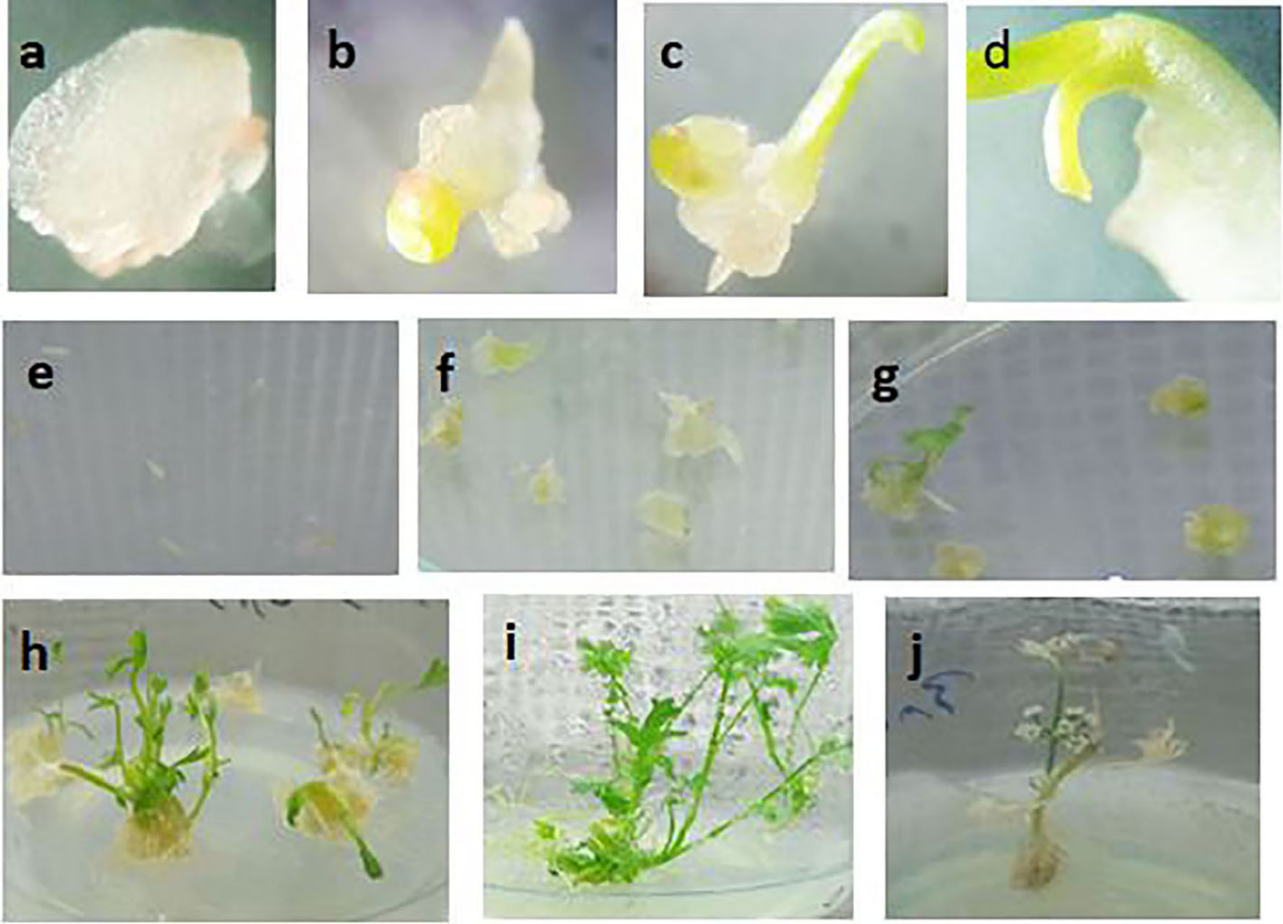
In vitro regeneration of *Trachyspermum ammi* using zygotic embryos. **a** Callus formation. **b** Callus development. **c** Monopolar regeneration. **d** Leaf emergence. **e** Isolated embryo on B5 media supplemented with 0.5 mg/l BA and 1 mg/l NAA. **f** Callus induction from mature embryos. **g** Shoot regeneration from an embryo-derived callus. **h** Elongation of shoot and root. **i** Regenerated plantlet from the zygotic embryo. and **j** The appearance of the inflorescence

Callus induction and regeneration of *T. ammi* were acquired in all tested treatments (zygotic embryos and different concentrations of plant growth regulators) on MS and B5 media. The B5 medium indicated abundant callus induction from days 8 to 10. The B5 media supplemented with 0.5 mg/l BA and 1 mg/l NAA were also observed to be the foremost treatment for callus induction. The MS media supplemented with 1 mg/l 2,4-D, and 0.2 mg/l KIN also demonstrated high callus induction (Table 1). Moreover, plant regeneration on B5 and MS media demonstrated a good rate with all treatments so that the range of regeneration changed from 41.1 to 92.6% (Table 1). Fresh and dry weights of calli also varied from 0.27 to 0.53 mg and from 0.018 to 0.034 mg, respectively. Furthermore, the height of the regenerated shoots was varied from 5.53 to 10.23 cm (Table 1). A comparison made between the treatments demonstrated that the highest percentage of regeneration for this ecotype (Shaheideh Yazd) was obtained in B5 medium with 0.5 mg/l BA together with 1 mg/l NAA. We only used this medium in plant transformation since this medium showed the best regeneration in tissue culture. Callus induction, regeneration, and growth of zygotic embryo have been shown in Fig. 1.

### Determination of threshold concentrations of kanamycin and *GUS* expression in transgenic plants

**Fig. 2:**
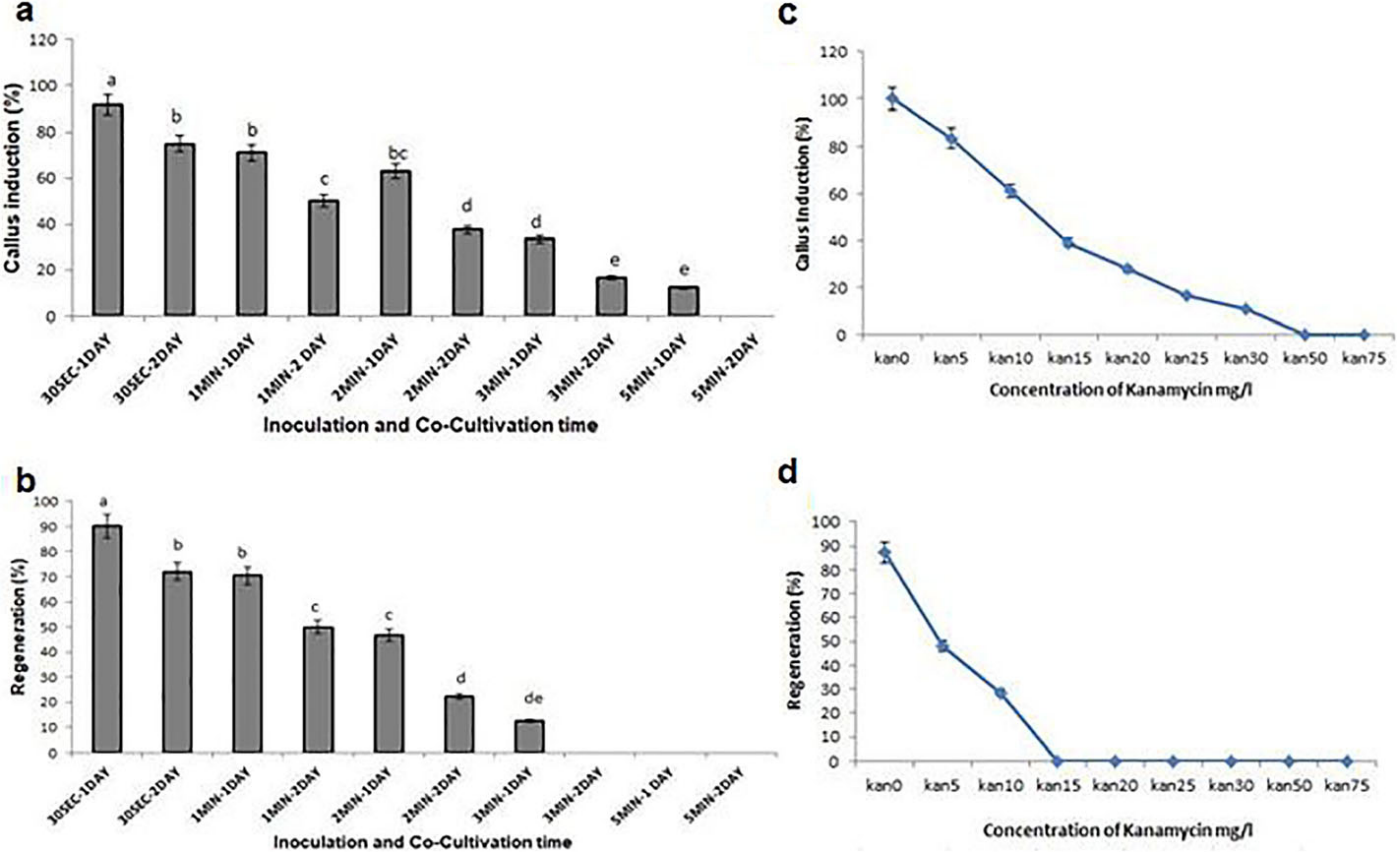
**a**, **b** Calluses’ induction and regeneration of *Trachyspermum ammi* by *Agrobacterium* LBA4404 harboring *GUS* gene based on inoculation time (30s, 1, 2, 3, and 5 min) and co-cultivation (1 and 2 days). To measure each treatment, all experiments were repeated up to three times. Different letters (**a**–**e**) specify a significant difference between treatments based on Duncan’s test at *P* ≤ 0.05. **c**, **d** The mean comparison of the percentage of callus induction and regeneration of *T. ammi* at different concentrations of kanamycin

**Fig. 3:**
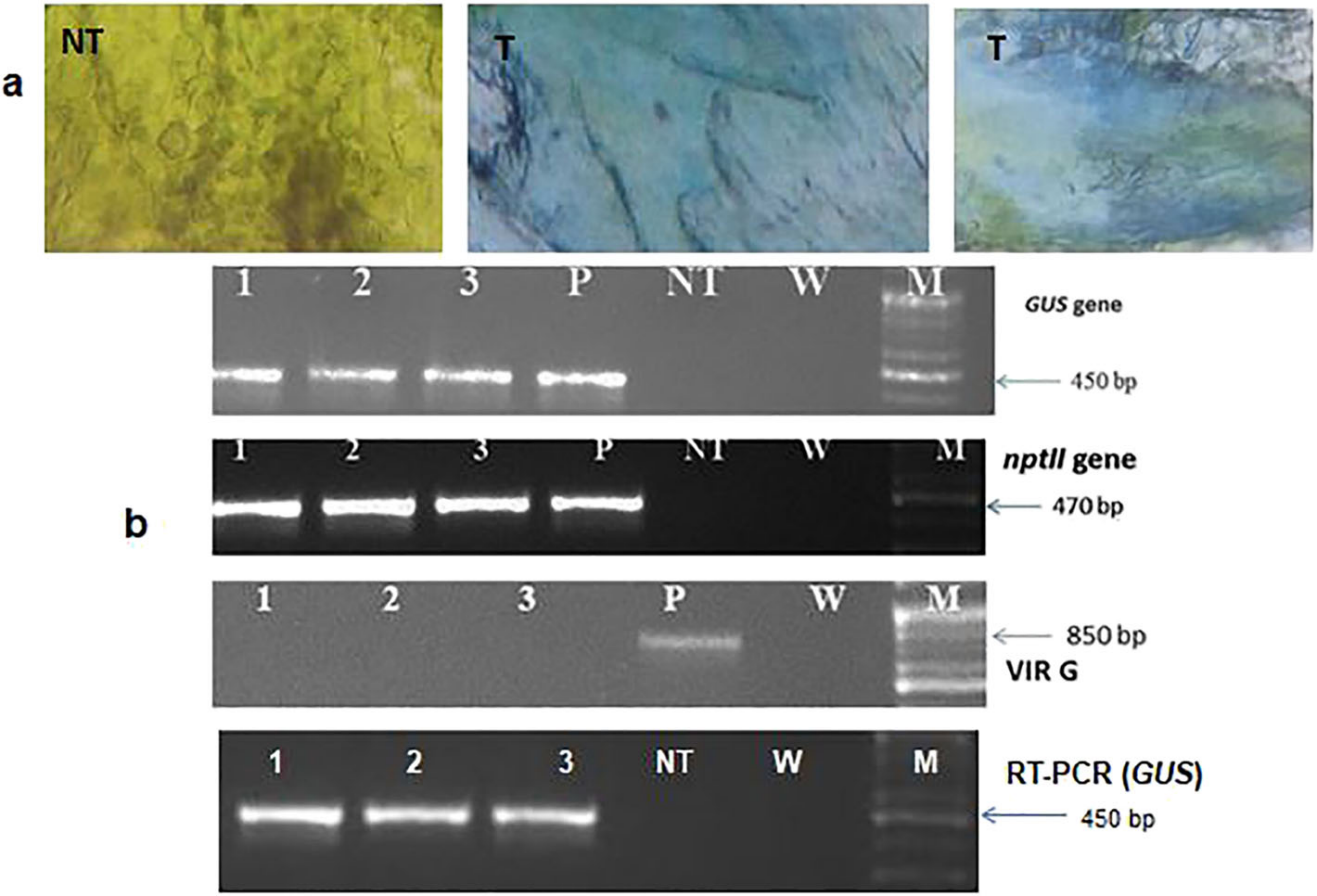
**a** Histochemical *GUS* assay using *Trachyspermum ammi* leaves, T: Putative transformed plant, NT: non-transformed plant (control); **b** Results related to PCR analysis of *GUS*, *nptII*, *VIR G*, and RT-PCR analysis. 1–3 putative transgenic plant, P: positive control (plasmid), NT: non-transformed herb, W: negative control, M: 100 bp DNA ladder

By studying the effect of kanamycin on the embryo culture, we noticed that calluses appeared in 0, 5, 10, 15, 20, 25, and 30 mg/l kanamycin but their initiation and growth were delayed by increasing kanamycin levels. Additionally, for higher concentrations of kanamycin, no callus induction was observed (Fig. 2c). Kanamycin concentrations (0, 5, and 10 mg/l) showed plant regeneration, and there was no regeneration at high concentrations of kanamycin (15, 20, 25, 30, 50, and 75 mg/l) (Fig. 2d). So, 15 mg/l concentration was applied as a threshold level of kanamycin for choosing the putative transgenic herbs. Findings the inoculation and co-cultivation time of LBA4404 harboring pBI121 with *GUS* gene also illustrated that 30 s inoculation along with 1-day co-cultivation had the most callus induction (Fig. 2a). Results of inoculation and co-cultivation time for regeneration showed that 30 s inoculation with 1-day co-cultivation had the most regeneration. These conditions were showed to be also appropriate for gene transferring process (Fig. 2b). Further, the existence of the *GUS* gene was confirmed with the presence of 450 bp fragments in 2% agarose gel. As it is observed in Fig. 3b, no PCR segment has been amplified in the non-transformed and the control sample (deionized water). The 470 bp segment has been amplified using the *nptII* specific primers (Fig. 3b). Also, to demonstrate the nonexistence of bacterial pollution, *VIR G*-specific primer was utilized. The fragments of 850 bp were obtained as a positive control using *VIR G* primer, and there was no any fragment for transformed plants on an agarose gel, confirming the nonexistence of *Agrobacterium* contamination in transformed plants (Fig. 3b). Results of histochemical *GUS* analysis, on the other hand, verified the existence of the blue color in transformed and no color in the non-transformed herbs (Fig. 3a). To approve the final expression of *GUS* in a transgenic plant, we carried out RT-PCR by *GUS*-specific primer. No bond existed in the control herb (Fig. 3b). Based on these results, the integrating the T-DNA harboring the *GUS* gene into the plant genome was verified. Hence, on the basis of the confirmation of gene existence in the plants and regenerating plants in the kanamycin culture medium, we can conclude that the regenerated plant is transgenic.

### *TPS2* expression in transgenic plants

**Fig. 4:**
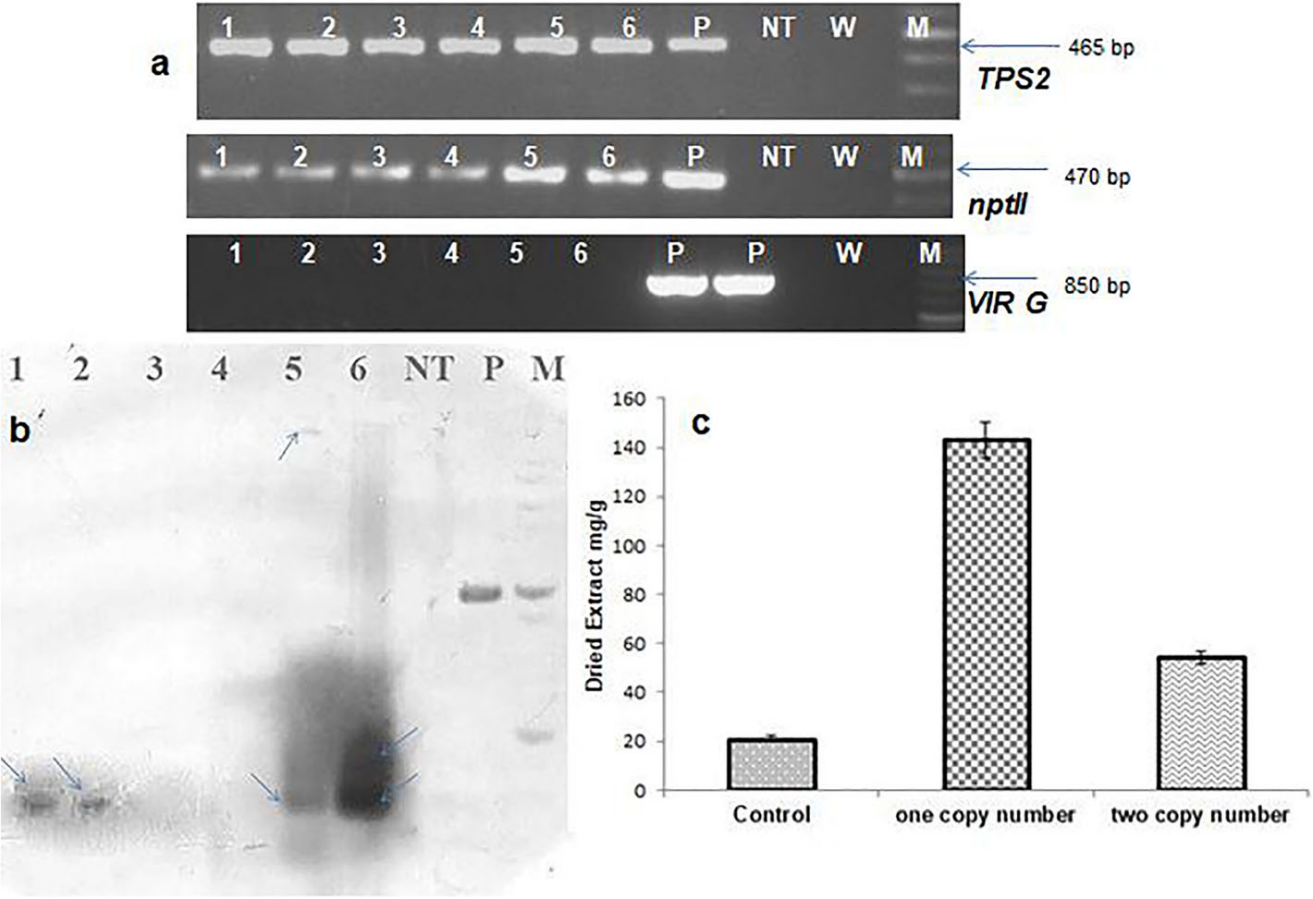
**a** Detection of putative transgenic *Trachyspermum ammi* by PCR 1–3: putative transgenic plant using LBA4404, 4–6: putative transgenic plants using EHA101; NT: non-transgenic plant; P: positive control (plasmid) W: negative control (deionized water); M:100 bp DNA ladder. **b** DNA-blot analysis of transformed and non-transformed *T. ammi*. 1, 2, 5, and 6 transgenic plants; NT: non-transformed plant; P: positive control; M: marker (digestion of total genomic DNA was performed using *Eco*RI). **c** Results of Thymol content (mg/g plant dried extract) of transformed and non-transformed *T. ammi* using HPLC methods. One copy or two copy numbers were determined using DNA-blot analysis

**Fig. 5:**
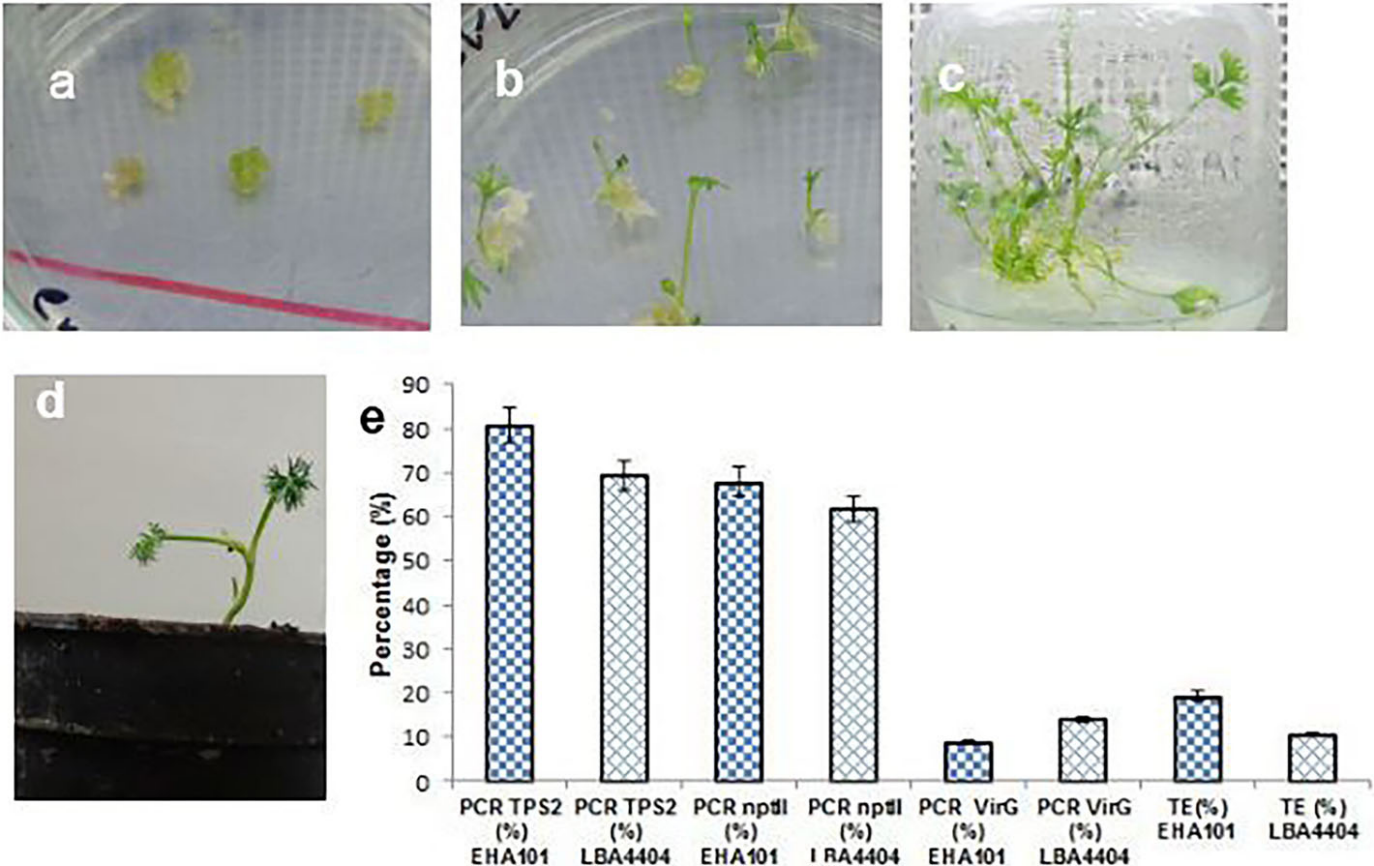
*Agrobacterium*-mediated transformation of *Trachyspermum ammi*. **a** Callus induction in selective media containing kanamycin and cefotaxime from zygotic embryos. **b** Development of shoot and root. **c** Putative transgenic plant. **d** Acclimatized transgenic plant in the pot. **e** Percentage of PCR results of transformed *T. ammi*, and TE: transformation efficiency

Meanwhile, a PCR amplification of 465 bp *TPS2* gene was proved in putative transgenic herbs (Fig. 4a). Following, the PCR was accomplished by specific primers of *nptII* and was verified with the presentation of 470 bp fragment on an agarose gel (Fig. 4a). PCR was also accomplished with *VIR G* primer to confirm lack of bacterial contamination. As a positive control, *Agrobacterium* indicated a fragment of 850 bp (Fig. 4a). Based on the outcomes of PCR, transformed herbs showed the transformation efficiency of 19.29% (EHA101) and 10.52% (LBA4404) (Fig. 5e). Moreover, six putative transgenic plants approved by repeated PCR analysis were analyzed via DNA hybridization to indicate the additional combination of the *TPS2* gene into the genome of transgenic herbs. Different models were observed for hybridizing in 4 out of 6 putative transgenic herbs. The results revealed one insert (two lanes 1 and 2) and more than one fragment (two lanes 5 and 6). No band was found in the non-transformed herb (lane NT) (Fig. 4b). Outcomes of HPLC of *Trachyspermum ammi* also indicated the highest thymol level in the transformed herbs. The thymol level was 143.90 mg/g, in the dried extract of transformed herbs (one copy number), 54.40 mg/g in the dried extract of transformed herbs (two copy number) and it was 21.09 mg/g in the dried extract of non-transformed herbs (Fig. 4c). These results showed 7-fold increment in thymol content (one copy number) and 2.5-fold increment (two copy number) than the non-transgenic plant. Several steps of regenerating the putative transgenic herbs are shown in Fig. 5a–d.

## Discussion

Numerous plants have potential medicinal uses and mainly contain valuable secondary metabolites representing anti-cancer, anti-inflammatory, antioxidant, and antimicrobial properties. The growing demand for these plant secondary metabolites suggests the use of biotechnology tools to produce transgenic plants in vitro. These methods have yielded valuable results and revealing that the production of transgenic plants is efficient and cost-effective to produce valuable secondary metabolite resources for medicine and industry [41].

Tissue culture methods are greatly applied to conserve and reproduce the medicinal plants that are laborious and time-consuming to propagate by conventional methods. Zygotic embryos are appropriate explants to regenerate plants because these explants, with convenient culture media, can produce many plants in the least time and with great proficiency. Different combinations of auxins and cytokinins were applied to regenerate *T. ammi*. Our results illustrated that the best treatment for callus induction and regeneration of plant was the B5 medium supplemented with 0.5 mg/l BA and 1 mg/l NAA. B5 medium with NAA and BA has been used to regenerate some plants from zygotic embryo explants [42, 43]. Results of our study illustrated that BA is necessary for regeneration by zygotic embryo. This has been supported by some previous studies [44, 45]. Moreover, the findings illustrated that zygotic embryo transformation was a suitable procedure for high regeneration and quick growth of *T. ammi*.

One of the most important ways to produce valuable plant secondary metabolites is to manipulate plant metabolic pathways by overexpressing or silencing selected elements in their biosynthesis pathway [41, 46]. Metabolic pathway manipulation is performed to increase the content of secondary metabolites (terpenoids) in the cultivation of many laboratory plants [47–49]. The results of this study showed that *the T. ammi* is transformed with both strains of *Agrobacterium* (transformation efficacy: EHA101 (19.29%), LBA4404 (10.52%). Hoseini et al. studied the transformation of *Arabidopsis thaliana* by the as part insulin gene and two *Agrobacterium tumefaciens* strains (EHA101 and GV 3101). They found that the EHA101 strain of *Agrobacterium* was more efficient than the GV3101 strain in gene transformation [50]. Pandey et al. [51] used two strains LBA4404 and EHA101 for the transformation of *Withania somnifera (L.) Dunal*. They demonstrated that LBA4404 had more *gusA* expression compared to EHA101. In our study, DNA hybridization was utilized for further verification of the combination of the *TPS2* gene into the plant genome. We found that the different dimensions of hybridization signals are caused by the stable T-DNA combination with the genome rather than endophytic *Agrobacterium* pollution. Moreover, according to HPLC analysis, a transgene copy can have a high potential for increasing gene expression in the transgenic *T. ammi*. Ma et al. found one to three copies of DNA hybridization fragments in transformed *Veratrum dahuricum* [52]. Dai et al. [53] reported that transgenic plants with more copy numbers of transgene showed lower level of *GUS* gene activity. It could be due to gene silencing mechanism. However, Alvarez [54] reported that copy number has no effect on foreign gene expression.

The production of high-capacity transgenic plants to produce valuable plant compounds creates a new field for discovering natural molecules, derived from both plants or plant-microbial interactions, for medical and other valuable purposes [55]. Vamenani et al. [56] showed that the transformation of *Ttrachyspermm ammi* by *Agrobacterium rhizogenes* strains (A4, LBA 9402, ATCC 15834) and seedling stem can increase thymol content (11.30 mg/g DM). This study demonstrated 5.3-fold enhancement of biomass and thymol agglomeration. In another study, to enhance drought and salinity tolerance in *T. ammi*, betaine aldehyde dehydrogenase gene and hypocotyl explants were used. The results showed enhancement thymol level in both wild type and transformed plants of ajowan (39.2 and 55.07%) by the drought stress [57]. In another study conducted by Sharma et al. [58], it was found that overexpression of Tryptophan decarboxylase and strictosidine synthase in *Catharanthus roseus* increased vindoline, catharanthine, and vinblastine content.

## Conclusions

In the current research, a fast protocol was introduced for the transformation of *Trachyspermum ammi*, using zygotic embryo explants within 25–35 days. The introduced protocol displayed the integration of *TPS2* gene with transgenic plants and the transformation efficiency 19.29%. Finally, HPLC analysis confirmed the increase of thymol in the aerial part of *T. ammi* due to expression of *TPS2* gene (one copy number). Overall, this study could be a practical protocol to enhance significant constituents in this plant; therefore, it can be beneficial for biotechnological research studies and pharmaceutical uses.

## Data Availability

Not applicable

## Abbreviations

MS: Murashige and Skoog
B5: Gamborg’s B5
BA: 6-Benzyladenine
NAA: 1-Naphthaleneacetic acid
2,4-D: 2,4-Dichlorophenoxyacetic acid
KIN: Kinetin
GUS: β-Glucuronidase
